# Phytotherapy in obstetrics – therapeutic indications, limits, and dangers

**DOI:** 10.25122/jml-2021-0353

**Published:** 2021

**Authors:** Corina Grigoriu, Valentin Varlas, Gina Călinescu, Andra Magdalena Bălan, Nicolae Bacalbașa, Consuela-Mădălina Gheorghe, Teodor Salmen, Corina Aurelia Zugravu, Roxana Elena Bohîlțea

**Affiliations:** 1. Department of Obstetrics and Gynecology, Carol Davila University of Medicine and Pharmacy, Bucharest, Romania; 2. Department of Obstetrics and Gynecology, University Emergency Hospital, Bucharest, Romania; 3. Department of Obstetrics and Gynecology, Filantropia Clinical Hospital, Bucharest, Romania; 4. Department of Marketing and Medical Technology, Carol Davila University of Medicine and Pharmacy, Bucharest, Romania; 5. Doctoral School of Carol Davila University of Medicine and Pharmacy Bucharest, National Institute of Diabetes, Nutrition and Metabolic Diseases N. C. Paulescu, Bucharest, Romania; 6. Department of Hygiene and Ecology, Carol Davila University of Medicine and Pharmacy, Bucharest, Romania

**Keywords:** phytotherapy, pregnancy, obstetrics, therapeutic indications

## Abstract

The wide access to varied, attractive, and aggressively promoted information can induce pregnant women to think that any form of complementary therapy can be a saving solution for a medical problem because these therapies are natural, therefore, harmless. Updated information from literature about indications, benefits, limits, and risks of phytotherapy in pregnancy was presented. Valuable therapeutic resources with proven clinical efficacy (evidence-based medicine) were presented for each trimester of pregnancy, during labor, postpartum, but also during breastfeeding. For some phytotherapeutics, there are scientific studies. There is also a detailed presentation about some possibilities for therapeutic errors, which should be avoided during pregnancy. Positive results of phytotherapy deserve to be known and applied by the obstetrician for the certain benefit of future mothers.

## Introduction

Wide access to varied, attractive, and aggressively promoted information can induce patients, especially pregnant women, to perceive that a certain type of complementary therapy can be a saving solution for a medical problem because these therapies are natural, so harmless. For an experienced clinician, there are sometimes medical situations in which it would be desirable to have “something else” as a therapeutic tool, to intercede for a better evolution of the patient when usual medicine seems to be inadequate. The various branches of complementary medicine, which never exclude modern medicine, can sometimes provide solutions. Pregnancy, the time of birth, but also the period of breastfeeding can be challenging for both obstetricians and patients and sometimes require a plus in the therapeutic panoply [1].

## Complementary Medicine in the Management of the Pregnant Patient

Complementary medicine consists of diagnostic and treatment methods outside modern, conventional, or classical medicine. An important part is represented by natural therapies (phytotherapy, gemmotherapy, aroma or oleotherapy, apitherapy, balneotherapy, hydrotherapy, and many others). Another important part is represented by the traditional medicine (Romanian, Asian, or from any other continent) to which, due to the intense circulation, many people have access: homeopathy, osteopathy etc. The strongest criticism of these methods is that therapeutic efficacy is not tested by scientific methods, and many experts are intrigued with the consumption of natural products that are increasing in the Western world, despite the absence of these scientific demonstrations of efficiency and/or safety. 

This article reviews exactly that branch of natural medicine that came beyond the tradition of folk medicine with scientific studies as a support for both clinicians and patients – phytotherapy. Phytotherapeutics use is estimated to be 10–74% of pregnant women (in Africa, Asia, Australia, Europe, and the USA) [1]. Also, during breastfeeding, about 30% of patients report that they use phytotherapeutics (for triggering/maintaining lactation or intercurrent diseases) [2]. Statistically, women are considered the largest consumers of phytotherapy, both for prevention and treatment [3]. Worldwide, the concern of obstetricians for herbal medicine is constant. The most respected journal of American obstetricians has very recently published a systematic review on the use of phytotherapeutics in pregnancy and postnatal periods [4]. After selecting valuable studies published in the literature, the author could draw conclusions on 47 phytotherapeutic products (on a cumulative population of 1,067,071 women). Also, scientific reviews of some phytotherapeutic preparations widely used in obstetrics – raspberry leaf, ginger, licorice, echinacea, essential oils, are published in some prestigious publishing journals [5].

### Phytotherapy

Phytotherapy is a branch of medicine that uses pharmaceutical preparations obtained from herbal products in prophylaxis and treatment. These preparations can be macerates, infusions, decoctions, tinctures, extracts, fresh or fermented juices, and powders. The origin of plant products is spontaneous flora, crop flora, industrial cell and tissue cultures, and the marine environment [6]. There are clear international rules and regulations on the harvesting, processing, and storing of plant products to prevent their degradation and contamination. Quality control of plant products is also provided in all national Pharmacopoeias, including the Romanian Pharmacopoeia. However, a debatable element is the fact that in our country, the preparations from herbal products are not registered at the National Medicines Agency, but either as food supplements at the Ministry of Health or at the Ministry of Agriculture.

We can distinguish between traditional phytotherapy (empirical, valuable over time, but without scientific evidence or clinical studies; traditional Romanian phytotherapy is very well represented at this part) and modern phytotherapy, which is based on scientific data of phytochemistry, pharmacognosy, and toxicology [7]. The therapeutic effect of plants is due to their active principles represented by some chemical constituents with pharmacodynamic activity, responsible for the pharmacological action of the plant. What is extremely interesting and different from synthetic drugs is that plants contain several active principles with different chemical structures and actions. All together form the so-called Phyto-complex. It is also very important that the various components of a plant product (e.g., leaves, flowers, roots, whole plant etc.) often act synergistically, the final therapeutic effect being potentiated in this way [6].

Regarding the scientific proof of the action of phytotherapeutic drugs, there are both clinical studies and reports of “evidence-based medicine”, but criticized when meta-analyzes are made due to either small groups of control or lack of double-blind experiments, or other error factors. However, there are multiple sources of documentation available for specialists. In this regard, the Drugs.com website contains data on phytotherapeutics in the Professional section. This is of interest to obstetricians because a small section of action in pregnancy and lactation is dedicated to each phytotherapeutic/plant, including the bibliographic source.

Of particular interest are preparations containing herbal combinations, which have multiple indications and whose components do not always have beneficial effects but also some toxic effects by accumulation. In case of these insufficiently verified “food supplements”, especially in pregnant or lactating women, these herbal combinations must be used with caution and only on the recommendation of a phytotherapy specialist.

The most harmless application of herbal therapy is by inhalation (in respiratory diseases) followed by infusion and then plant extracts. The most hazardous active substances in pregnancy are alkaloids, coumarins, saponins, cucurbitacins, sesquiterpene lactones, and anthraquinones.

It should be noted from the beginning that some phytotherapeutics are prohibited during pregnancy or should be used with caution due to the emmenagogue effect (which means the stimulation of uterine contractility, so the risk of miscarriage premature birth). Plants with a laxative and diuretic effect are also prohibited [6].

The plants with the emmenagogue effect are common yarrow (*Achillea millefolium*), virnant (*Ruta greveolens*), sage (*Salvia officinalis*), mother root (*Leonuri herba*), sweet flag (*Acorus calamus L*), but also marigolds (*Calendula officinalis*), licorice (*G glabra*), parsley (*Petroselinum crispum*), thyme (*Satureja hortensis L*), fennel (*Foeniculum vulgare*), wormwood (*Arthemisia absinthium*), Syrian weevil (*Hybiscus syriacus*), horsetail (*Tussilago farfara*), verbena (*Verbenna officinalis*), senna (*Senna alexandrina*), buckthorn (*Rhamnus frangula*), nettle (*Urtica dioica*), juniper (*Juniperus communis L.*), lavender (*Lavender Angustifolia*), anise (*Pimpinella anisum*), and gentian (*Gentiana lutea*).

Plants banned in pregnancy primarily due to the embryotoxic effect of alkaloids are dragonfly (*Berberis vulgaris*), hollyhock (*Chelidonium majus*), carrion (*Ephedra sp*), Hydrastis (*Hydrastis Canadensi*s). Due to the bitter substances, essential oils, and the emmenagogue effect, the following plants are forbidden in pregnancy: aloe vera (*Aloe vera*), hot pepper tincture (*Capsicum annuum*), sorrel (*Symphytum officinale*), thimble (*Pulsatilla sp*), mistletoe (*Viscum album*), juniper, and cinnamon essential oil (*Cinnamonum verum*) [5].

Certain herbs used as spices should be avoided or consumed occasionally and, of course, in small quantities (basil, cumin, parsley, larch, oregano, horseradish, rosemary, marjoram, and saffron). The plants of Asian medicine forbidden in pregnancy are the dwarf palm (*Serenoa repens*), Dong-Quai (*Angelica Sinensis*), and yohimbine. Some plants are allowed only for occasional consumption: hyssop, St. John’s wort, burdock, plantain, armory, fenugreek, and thyme.

Pregnant patients may use preparations (simple infusions or mixtures) containing the following plants: chamomile, mint, raspberry leaves, rosehip, ginger, cranberry, blueberry, rhubarb, and echinacea, which are allowed in reasonable quantities and have no side effects [8].

There are countless combinations of tea or tablets indicated for pregnancy, but their composition must always be carefully checked. If these products are consumed in larger quantities than recommended, the side effects may occur (most often exaggerated uterine contractility) [9]. In Romania, for example, a tea for pregnant women is marketed containing rooibos (*Aspalathus linearis*), raspberry leaves, nettle, ginger, alfalfa (*Medicago sativa*), lime fruit, parsley (the plants highlighted are emmenagogues). There is also another “tea for pregnant women”, which contains rosehip (*Rosa canina*), elder (*Sambucus L.*), pear root (*Elymus repens*), wild pansy (*Viola tricolor*), dandelion (*Taraxacum officinale*), linden (*Tilia tomentosa*), blueberry leaves (Vaccinium myrtillus) – indicated in “pregnancy edema”, an extremely complex clinical entity, which can hide a dangerous pathology, both for mother and fetus. It must be correctly diagnosed and treated. In terms of preparations containing ginger (*Zingiber officinalis*), there is a highly varied offer, especially in terms of doses (tablets with ginger 185 mg, tablets containing vitamin B6 2 mg and dry extract of ginger rhizome standardized in gingerol 16 mg, the recommended dose beings 2 to a maximum of 8 tablets per day, 550 ginger capsules, the recommended dose beings one to six capsules per day, capsules with standardized ginger root extract, 250 mg, guaranteed 5% gingerol (12.5 mg), one capsule per day being recommended.

On the other hand, not every combination containing ginger extract is allowed in pregnancy:

• Ginger 300 mg, wild peppermint essential oil 0.5 mg, rhubarb essential oil 0.5 mg – tablets (ingestion of essential oils is not permitted);
• Ginger 160 mg, artichoke, yellow gentian (*Gentiana lutea*). Although the preparation contains gentian, which is contraindicated in pregnancy, the leaflet in the chapter “Contraindications” states that they are unknown.

### Gemmotherapy

An important branch of phytotherapy, in continuous development, is represented by gemmotherapy, which uses the youngest plant tissues – meristems, buds, branches, or roots. The plant is renewed from these embryonic tissues, all these tissues having an anabolic effect, reproductive capacity, and cell multiplication. Glycerin macerates are made from these components of the plant [10]. The preparations administered are hydro-glycero-alcoholic solutions in dilution 1 to 10 (1DH). Some gemmotherapeutics are contraindicated in pregnancy, namely: extracts of black and white alder, boxwood, peach, apricot, almond, mistletoe, juniper, white willow (mint), and Tamarix.

Raspberry (*Rubus idaeus*) is the most valuable gemmotherapeutic remedy for women during the reproductive period and perimenopause. Buds and branches are characterized phytochemically. They contain organic acids, vitamins, and catechins, which have an anti-inflammatory and antioxidant effect. Catechins (13.25%), flavonoids (8.71%), cinnamic acid, and benzoic acid predominate. Its indications are habitual abortion or the risk of abortion (association of raspberry, cuddly, and hornbeam hemoderivatives) and the risk of premature birth [11].

The gemmotherapeutic from crab (*Malus silvaticus*) acts on the hypothalamic-pituitary-adrenal gland and ovarian axis, having a progesterone-like effect [12]. It is administered in pregnancy in the first three months when deciduous hematomas appear. It is used as a progesterone supplement, but also in the last weeks of pregnancy, having a favorable effect on heartburn (together with the gemmoderivative from the fig).

The gemmoderivative from viburnum (*Viburnum lantana*) can be used in obstetrics: the risk of abortion or premature birth and can be prophylactically administered to the patients with abortive disease when the pregnancy is detected.

The blackcurrant hemoderivative (*Ribes nigrum*) is one of the most studied gemmotherapeutic [13]. It acts like cortisone: it is an acute phase anti-inflammatory, with an important antiallergic effect, an immune stimulator, indicated in local and general inflammatory syndromes, acting mainly in the mucous membranes (respiratory, urogenital, digestive) [14, 15].

The hemoderivative from linden (*Tilia tomentosa*) affects the neuro-vegetative system, being useful in pregnancy in case of insomnia and anxiety, being preferably administered in the evening.

### Aromatherapy

Aromatherapy is a branch of phytotherapy that treats diseases by using volatile oils called aetherolea (improperly called “essential oils”) extracted from plants. Volatile oils (aetherolea) are products of secondary plant metabolism secreted by specialized cells. Volatile oils are stored outside the plant (in glandular brushes or papillae) or inside the plant (in secretory channels or bags) in the form of oily, volatile, aromatic-smelling liquids [6]. In the broadest sense, aromatherapy refers to the external use of volatile oils by inhalation, baths (warm 28–35 degrees Celsius), sauna, massage, reflexology. Clinical aromatherapy means the internal administration of aromatic oils. Internal administration requires rigorous control of oil quality and standardization (determination of organoleptic characteristics, density, specific rotational power, refractive index, acidity index, ignition temperature, and boiling temperature). At present, there is a very wide variety of volatile oils for sale, with varying indications of use, with varying names (aromatic oil/essential oil etc.), and certainly of varying origin and quality. The quality of a volatile oil depends on the plant from which it is obtained (botanical origin, plant organ, vegetative cycle), geographical area (pedoclimatic peculiarities), seasonal conditions (season, temperature, precipitation, exposure to sunlight), and finally, the influence of the phytosanitary treatment (herbicides, insecticides) or fertilizers [6].

Volatile oils used for medical purposes must be of high quality. Unfortunately, many synthetic or counterfeit oils do not have the expected therapeutic effects or can even be dangerous. Therefore, the source and the producers of volatile oil must be rigorously checked. In pregnancy, volatile oils are administered strictly externally by massage or inhalation (partial baths, aromatherapy lamps, application on a napkin) [16]. The following products have an absolute contraindication in pregnancy: anise essential oil, star anise, dill, fennel, hyssop, basil, juniper, nutmeg, and Spanish lavender. Likewise, oils with toxic effects on the central nervous system are also not used in pregnancy: oils containing ketones (thuja and sage) and eucalyptus (proconvulsant effect) [17].

## Discussion

From the data published in the literature and personal clinical experience, we discussed the indications of the main phytotherapeutics used in obstetrical practice, possible interactions with various drugs, side effects, and plants contraindicated in pregnancy [18].

### Examples of phytotherapeutic drugs with obstetric indications

#### Raspberry (Rubus idaeus) leaves

Indications are preparation of birth by stimulating the uterine contractility, having an indirect hemostatic effect in the immediate postpartum. It is also indicated in term labor induction or post-term pregnancy [4]. Although many studies show that the infusion of raspberry leaves is the first one used as a recommendation for pregnant women, there is little scientific evidence of its mechanism of action and effectiveness. Experimental studies on fragments of non-pregnant and pregnant uterus on both animal and human tissues prove a stimulation of the contractility of the human pregnant uterus, but many results are contradictory [4].

We cannot draw clear conclusions on the beneficial effect (shortening the second phase of labor by ten minutes, decreasing the use of forceps) from clinical trials. The therapeutic dose appears to be four grams/day, and the only randomized, double-blind study used 2.4 g/day, which may explain the lack of significant conclusions. Based on meta-analyzes, a scientifically based recommendation for the use of raspberry leaves in pregnancy cannot be made at this time [5].

#### Ginger (Zingiber Officinalis)

It is used mainly at the beginning of pregnancy, in the first trimester dysgravidia, and the last weeks, when a digestive discomfort may appear [19]. We recommend either fresh ginger or dry ginger (powder), extract, or essence. The maximum allowable dose is 10 g dry ginger (or 30 g fresh ginger)/day. The mechanism of action would be through the action of gingerol and shogaol as antagonists of cholinergic M3 receptors and serotonergic 5-HT3 receptors localized in the central nervous system, but also through the gastro-tonic effect, increased gastric motility, and evacuation of the stomach by peripheral anticholinergic and antiserotonergic actions [20]. Meta-analyzes verified 14 randomized trials, observing over 70,000 pregnant women. The only side effects consistently reported in meta-analysis studies are digestive (burns in the epigastrium). There is still a question mark regarding the antiplatelet effect in pregnancy. No higher rates of miscarriage, premature birth, fetal death, or malformations were observed. Thus, the use of ginger seems to be safe for both mother and fetus. The quality of the preparations used, and any incorrect combinations of the producers should be discussed. Currently, no standard dose can be recommended, but a dose of more than 1 g/day for at least four days seems to be effective. The most active component of the preparations is 6-gingerol (its concentration should be reported by the manufacturer), and the toxic substance is methyleugenol.

#### Bryophyllum pinnatum (tropical plants represent genus Kalanchoe)

Bryophyllum extract has a tocolytic effect, being used in Germany in abortion or premature birth [21]. The peculiarity is the absence of adverse fetal reactions compared to betamimetics or other tocolytics [22]. The form of administration is by intravenous infusion, 5% solution for injection, four 10 ml vials in 1000 ml saline. The extract improves the tense mental state of pregnant women, reducing nervousness or agitation [23]. An anthroposophic preparation, made of Bryophyllum extract, silver, and a potentiated preparation from the uterus – Bryophyllum compose – globules and vials – help a premature birth and the nervous dysfunctions associated with premenstrual syndrome or perimenopausal neurovegetative disorders.

### Examples of interactions with various drugs

As pregnant women generally receive classical medication for various pathological situations, and at least one in five pregnant women consumes natural products simultaneously, the potential interactions between them must be considered [24].

• Ginger – possible interactions with Insulin, Metformin, Nifedipine;
• Chamomile – possible interactions with benzodiazepines (increases the central nervous system suppressing the effect of drugs) and Ondansetron (decreases the antiemetic effect);
• Raspberries – it is discussed that polyphenols may decrease the intestinal absorption of iron, but there is no solid scientific evidence in this regard.

### Examples of side effects

• Gastrointestinal effects (soft stools, gastrointestinal irritation, diarrhea) – capsaicin from hot peppers (study in pregnant women with gestational diabetes); gastroesophageal reflux, abdominal discomfort, increased nausea – ginger (first-trimester dysgravidia) [18];
• Premature birth (oral – excessive consumption of chamomile tea; licorice (high consumption, over 500 mg/week, in the form of candy); topical – almond oil, for massage (but the direct causal link could not be demonstrated);
• Preeclampsia – consumption of licorice in the second trimester;
• Maternal hypoglycemia – Raspberry leaves (used as an inducer or stimulator of labor) [25];
• Polyhydramnios – senna leaves;
• Hepatotoxicity – preparations from plants contaminated with lead, senecionine;
• Malformative effects – however, the correlations are weak: Angelica Sinensis used to prevent abortion could induce musculoskeletal and connective tissue malformations or ocular malformations [18].

Various phytopreparations have also been studied, no adverse reactions appear to have been recorded, but the quality of these studies is low to mediocre. Therefore, caution is recommended for the following substances: evening light capsules or saffron or dill infusions to induce labor, red sage for oligohydramnios, ginger, or quince for first trimester dysgravidia, ointments with green tea extract to heal the episiotomy scar.

### Examples of the limits of phytotherapy

Excessive consumption of fennel by lactating women can lead to increased absorption of anethole (phytoestrogen) and estragole (etheric component). Estragole, which is also found in many plants and spices (basil, tarragon), has been shown to have a carcinogenic effect in mice (at a dose of more than 1 g/kg body weight) [26, 27]. Excessive use of fennel can trigger postpartum hemorrhage. Turmeric, known for its beneficial effects outside pregnancy, is not sufficiently studied so that no recommendations can be made regarding its use during pregnancy.

### Therapeutic schemes applicable to pregnant patients

Based on scientific data from literature and meta-analyses, several therapeutic schemes can be recommended for the most common pathologies in pregnancy, some of them with scientific documentation.

#### Threatened abortion

• Phytotherapy: The infusion of Lady’s mantle (*Alchemilla vulgaris*), one or two cups a day, or passion flower (*Passiflora incarnata* or *Passiflora caerulea*) are useful. Traditional Chinese medicine recommends Angelica sinensis;
• Gemmotherapy: gemmoderivative from raspberries, crab (progesterone-like effect), viburnum, and hornbeam (antispasmodics, muscle relaxants).

#### First trimester dysgravidia

• Phytotherapy: infusions in small quantities, even cold ones, from the following plants: ginger, mint (weak infusion), hop (*Humulus lupulus*), chamomile (*Matricaria recutita*), fennel;
• Gemmotherapy: fig-glycerol-alcohol maceration, 2 ml twice a day;
• Aromatherapy: essential oil from ginger, lavender, lemon, rosewood, rhubarb, and mint (external use only).

#### Insomnia

• Phytotherapy: mild sedative infusions of linden flowers, valerian (*Valeriana officinalis*), hop cones, lavender, or rhubarb (*Melissa* *officinalis*) (simple or mixtures);
• Gemmotherapy: lime extract.

#### Digestive disorders (gastroesophageal reflux, constipation, diarrhea)

• Phytotherapy:
  – gastroesophageal reflux: infusion of ginger, chamomile, mint, fennel [28];
  – pregnancy constipation should not be combated with laxative teas because most of them will stimulate contractility (the common infusion of chamomile consumed for this purpose presents a risk for miscarriage or premature birth). However, mild laxatives – flax seeds or certain laxatives of volume -psyllium) may be administered;
  – diarrhea: infusion of dried blueberries.
• Gemmotherapy:
  • in gastro-esophageal reflux and heartburn: fig extract 2 ml x 2/day;
  • cranberry extract in constipation.

#### Hemorrhoidal disease

• Phytotherapy: creams with poplar buds, hamamelis, oak bark or suppositories with marigold flower extract, hamamelis, poplar buds, chestnut seeds may be recommended;
• Gemmotherapy: extract of wild chestnut and edible chestnut.

#### Venous pathology in pregnancy

Phytotherapy: for the prophylaxis of varicose veins, infusions of nettle, hamamelis, Lady’s mantle combined, one cup daily are recommended. There are many gels, creams, or capsules with known plant extracts with venotropic effects (wild chestnut, vine, hamamelis). Oral preparations containing standardized extracts of Citrus Aurantium, containing 500 mg of micronized purified flavonoid fraction corresponding to 450 mg of diosmin and 50 mg of hesperidin, extracted from citrus peel, may be used from the second trimester of pregnancy).

#### Anemia

• Phytotherapy: infusion of nettle, dandelion, elder juice.

#### Urinary tract infections in pregnancy

• Phytotherapy: blueberry infusions, cranberries. Many over-the-counter preparations contain blueberry extracts, American blueberry (*Vaccinium macrocarpon*), cranberry (*Vaccinium Vitis-idaea*), goldenrod (*Solidago virgaurea*);
• Gemmotherapy: blueberry derivative, cranberry, green figwort.

#### Allergies in pregnancy

• Gemmotherapy: 2 ml x 3 per day blackcurrant gemmoderivative or compound preparations, local solutions, administered by inhalation, from gemmotherapeutic extract from blackcurrant buds and dried Astragalus membranaceus root extract.

#### Labor

• Phytotherapy: The phytotherapy of choice is Lady’s mantle (*Alchemilla Vulgaris*, infusion and tincture or combination), known in traditional European phytotherapy, which optimizes uterine contractility. Infusions of yarrow plant, raspberry leaves, verbena, blackberry leaves (*Rubus fructicosus*), and Cimicifuga can be added;
• Aromatherapy: its use seems to reduce fear, anxiety (especially primiparous), pain, nausea in labor, and increase uterine contractility. Thus, the essential oil of lavender, jasmine, lemon, and rose (*Rosa damascena*) reduces fear, the essential oil of sage (*Salvia sclarea*) stimulates contractility but calms the parturition. Cinnamon leaf oil also stimulates uterine contractility. Studies show a shortening of the active phase of labor but not a decrease in its entire duration. The third period of labor is shortened. Jasmine essential oil calms the perception of painful uterine contractions, but also galactagogue.

#### Breastfeeding

• Phytotherapy: cumin, anise, or fennel (but for moderate consumption, as infusions, three cups of tea a day). It should be noted that the intake of these teas is not a substitute for proper hydration, so consumption should be moderate. Tea made from mallow, chamomile, and rhubarb flowers can also be combined;
• Aromatherapy: Essential oil from lemongrass (*Cymbopogon citratus*) restores the mother’s body after birth, stimulates lactation, fights postnatal depression, sweet cumin (fennel), sage, and jasmine.

#### Weaning

• Phytotherapy: mint and sage tea, preparations from *Vitex Agnus Castus*. Plants that decrease or stop lactation must be known: sage, thyme, yarrow plant, nettle, sorrel (infusion), local applications of cabbage (crushed leaves), jasmine (flowers), and parsley (leaves);
• Aromatherapy: essential oil of cypress, geranium, lavender, mint (compresses with 2–3 drops in 20 ml of vegetable oil, applied to the breasts).

#### Mastitis

• Phytotherapy: poultice from concentrated infusion of thyme, which is kept for one hour a day on the inflamed area of the breast; poultice from crushed fresh cabbage leaf (but applied strictly on the inflamed area, because it can stop milk secretion);
• Aromatherapy: chamomile, geranium, lavender, mint – compress applied to the inflamed area.

#### Pain control after cesarean section

Adding aromatherapy (lavender, jasmine, lemon and rose) to analgesia [29] appears to decrease anxiety and pain perception 12 and 24 hours after cesarean section, but the authors are not sure that the effect would be different from placebo [30].

#### Healing scars

• Phytotherapy: locally, for episiotomy wounds, concentrated infusions of wormwood, horsetail, marigold, chamomile, combined with antiseptic and healing creams of marigold and arnica;
• Aromatherapy: chamomile oil, lavender, tea-tree, cypress, incense, myrrh.

#### Postpartum depression

• Phytotherapy: reasonable amounts of St. John’s wort infusion or St. John’s wort extract may be recommended;
• Aromatherapy applied in labor and postpartum (lavender, jasmine, lemon, and rose) seems to decrease the incidence of postpartum depression.

#### Postpartum recovery

• Phytotherapy: general tonics such as fruits of Prunus spinosa, fig, or dates fruits paste;

#### Intercurrent respiratory disorders in pregnancy

• Phytotherapy: infusions of lime, mint, lemon, rosehip, echinacea, ginger [31–33];
• Gemmotherapy: combinations of blackcurrant, hornbeam, rosehip, and silver birch bud extracts.

#### Covid 19 infection during pregnancy

• Phytotherapy: infusions of rosehip and echinacea, which are associated with an extremely favorable effect. Apitherapy (prophylactic and curative – therapeutic combinations of honey, pollen, and royal jelly);
• Aromatherapy: essential oil of fir, pine, lemon (antiseptics); lavender or bergamot essential oil (anxiolytics).

Even if complementary therapies have been proven beneficial for pregnant women, they should not be applied without care because they can sometimes do more harm than good. Some examples of this are lower limb edema, hypertension in pregnancy, preeclampsia [34], gestational diabetes, constipation – in which case the use of even “trivial” infusions can be extremely dangerous [35].

## Conclusions

Positive results of many complementary therapies deserve to be known and applied by the obstetrician, benefiting the future mother. On the other hand, few systematic reviews highlight the adverse effects of this therapy. Pregnant women can be supported to use these therapies during pregnancy rationally and while breastfeeding if indicated by a specialized doctor with experience in phytotherapy. The obstetrician and the family physician or pharmacist must encourage a pregnant woman to ask for special information. Also, pregnant women must not use self-diagnosis and self-medication in any form during pregnancy because the mistakes can be extremely severe, both for the mother and the fetus.

## Acknowledgments

### Conflict of interest

The authors declare that there is no conflict of interest.

### Authorship

CG and REB contributed to conceptualizing, VV and TS contributed to the methodology, CG and GC contributed to writing the original draft, AMB, TS, and NB contributed to editing the manuscript. CMG and CAZ contributed to data collection. CAZ and REB contributed to data analysis. 
